# Providing geriatric oncology care using telemedicine for older patients with cancer during the COVID-19 pandemic in Mexico

**DOI:** 10.3332/ecancer.2023.1528

**Published:** 2023-04-03

**Authors:** Celia G Hernández-Favela, Maria Renee Jimenez-Sotomayor, Andrea Morales-Alfaro, Andrea de la O-Murillo, Enrique Soto-Perez-de-Celis

**Affiliations:** Department of Geriatrics, Instituto Nacional de Ciencias Médicas y Nutrición Salvador Zubirán, Tlalpan, CDMX 14080, México

**Keywords:** older adults, telemedicine, cancer, developing countries, smartphones

## Abstract

The objective of this study was to describe our experience using readily available telemedicine tools to deliver specialist multidisciplinary care to older adults with cancer at a Mexican medical centre during the COVID-19 pandemic. Between March 2020 and March 2021, patients aged ≥65 years with colorectal or gastric cancer treated at a geriatric oncology clinic in Mexico City were included. Patients were reached via telemedicine utilising readily available apps such as WhatsApp or Zoom. We performed interventions such as geriatric assessments, treatment toxicity assessments, physical examinations and treatment prescription. The number of visits per patient, type of device used, preferred software/app, consultation barriers and the ability of the team to deliver complex interventions were analysed and reported. A total of 44 patients received at least one telehealth visit, with a total of 167 consultations. Only 20% of patients had webcam-equipped computers, and 50% of visits were performed using a caregiver’s device. Seventy-five percent of visits took place using WhatsApp, and 23% using Zoom. The average visit lasted 23 minutes, with only 2% not completed due to technical issues. A geriatric assessment was successfully conducted in 81% of telemedicine visits, and chemotherapy was prescribed remotely in 32%. The use of telemedicine is possible in older adults with cancer living in developing countries and with little previous exposure to digital technology using readily available platforms such as WhatsApp. Healthcare centres in developing countries should make efforts to enhance the use of telemedicine, particularly for vulnerable populations such as older adults with cancer.

## Introduction

Over half of new cancer cases worldwide occur in adults aged ≥65 years [1]. However, older adults still face many barriers for accessing age-friendly multidisciplinary oncology care [2]. Telemedicine is defined as the use of telecommunication technologies to provide health services at a distance and may represent an effective approach to mitigate existing barriers for accessing cancer care for older adults. Geriatric oncology care requires multiple interactions with healthcare personnel, which become increasingly challenging with ‘traditional’ healthcare provision, making telemedicine an attractive option [3]. During the COVID-19 pandemic, when access to health services was limited, telemedicine became an increasingly used option for the care of chronic conditions [4].

The American Society of Clinical Oncology (ASCO) has published telemedicine guidelines, highlighting its use for long-term treatment and supportive care, as well as for acute care to avoid emergency department visits and manage treatment [5]. Telemedicine may improve access to health services and reduce costs by allowing faster access to medical care. Likewise, telemedicine may increase equity of access to cancer care services and improve overall quality of care [6].

Misconceptions regarding technology use among older adults have slowed the implementation of telemedicine in geriatric oncology. However, the use of new technologies among older individuals has grown markedly, with 61% of US older adults owning smartphones, for example [7]. In Mexico, although <50% of the population owns a computer, >75% owns a smartphone [8]. While older adults are significantly less likely to own such devices, the penetrance of mobile technology and the Internet among older individuals in developing countries has increased, making this an attractive option for increasing the reach of healthcare systems with limited resources [9].

The implementation of telemedicine programmes for older individuals with cancer in developing countries requires an understanding of the availability of technological resources, as well as of the ability to undertake complex assessments and interventions remotely. Here, we describe our initial experience providing specialised multidisciplinary cancer care to older adults with cancer in Mexico utilising readily available telemedicine tools.

## Methods

This is a retrospective analysis of prospectively collected data of telemedicine visits for patients aged ≥65 years diagnosed with colorectal or gastric cancer at the geriatric oncology clinic at *Instituto Nacional de Ciencias Médicas y Nutrición Salvador Zubirán* (INCMNSZ)*,* a third level hospital in southern Mexico City providing care for uninsured patients. Visits occurred between March 2020 and March 2021, when INCMNSZ was converted into a COVID-19 centre.

Telemedicine visits started on 23 March 2020. Patients with follow-up appointments were contacted ≥24 hours beforehand via a phone call or text message and informed that their visit would occur remotely. Patients who came for a first-time visit and those who had disease progression or other issue which required the delivery of bad news were not scheduled for telemedicine visits and received usual in-person appointments. Patients were asked about availability of a computer, tablet or smartphone, as well as of videoconferencing software/apps (Zoom or WhatsApp). Patients were given a precise date and time for their consultation and advised to be ready with their device in a quiet place. Patients who chose to use Zoom videoconferencing software were sent a link using an email provided by the patient at least an hour before the appointment, while those who chose WhatsApp were called using the videocall function of the app at the time of the appointment. Family members could join in person at the patient’s home or using the same software in their own devices at the patient’s discretion. On the day of the consultation, patients were contacted using the preferred software and asked to turn on the video. Other team members, including a gerontologist with training in oncology, also joined the calls. Thirty minutes were dedicated to each patient. In cases of connectivity problems, the consultation could be changed to another software/app, changed to a phone call consultation or postponed. Assessments and interventions during the call included geriatric assessments, treatment toxicity assessments, physical inspection using the device’s camera, supportive care recommendations and treatment prescription (including chemotherapy). The remote geriatric assessment included evaluations of activities of daily living, nutritional status, depression, falls, sensory impairments and polypharmacy using validated tools. Telemedicine encounters were recorded in the electronic medical record. At the end of the appointment, prescriptions for medicines were sent via email or the WhatsApp app in a printable PDF format with institutional letterhead and signed by the treating oncologist. Future medical appointments, laboratory appointments for blood draws, chemotherapy suite appointments and imaging studies were arranged by the medical team using the electronic medical record, and the forms required for accessing such services were sent as a PDF files.

The following variables were recorded: number of visits, device utilisation, preferred software/app, encountered barriers and ability to undertake complex interventions such as geriatric assessments or chemotherapy prescriptions. Descriptive statistics were utilised to analyse the results of the study. The study was approved by the Ethics Committee at INCMNSZ and, since the provided services were standard of care, informed consent was waived.

## Results

Forty-four patients with a median age of 75 years (range: 65–89 years) received at least one telemedicine consultation during the study period and were included in this study. The total number of consultations was 167, with a median of three per patient (range: 1–9). Thirty-five patients (80%) had a colorectal cancer (29% Stage IV), while nine (21%) had gastric cancer (66% Stage IV). Thirteen patients (30%) lived outside the Mexico City Metropolitan Area. During the study period, 3 patients were diagnosed with COVID–19, and 13 died (30%).

Only nine patients (20%) owned a webcam-equipped computer, and 50% of visits took place using a device (smartphone or computer) belonging to a relative/caregiver. Seventy-five percent of consultations were performed using the WhatsApp app (*n* = 126), 23% were conducted via Zoom (*n* = 39) and only 1% (*n* = 2) were via telephone. The median consultation length was 23 minutes (range: 10–36 minutes).

**Figure 1.**
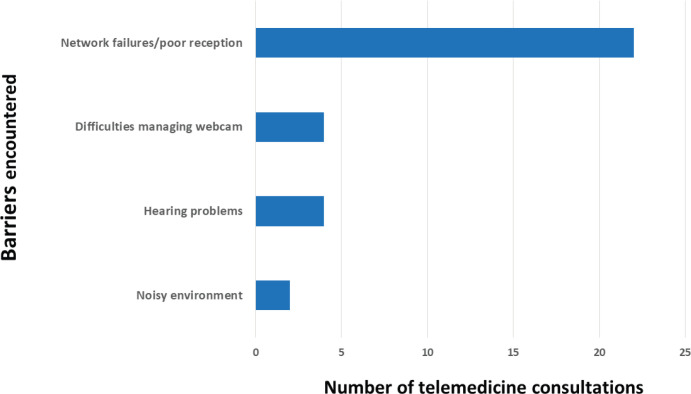
Barriers encountered during telemedicine consultations (*n* = 167).

Only four telemedicine consultations (2%) could not be completed in the allotted day due to technical issues and had to be postponed or conducted via telephone. None of the visits had to be converted to an in-person appointment. Minor issues which did not lead to cancellation occurred in 30 visits (*n* = 18%) (Figure 1).

Other team members participated in >80% of consultations. A gerontologist was present in 130 consultations (78%), while a physician assistant was present in 123 (74%). A geriatric assessment was performed in 135 consultations (81%), and chemotherapy was prescribed remotely for 14 patients (32%) in 53 visits after a review of toxicities. Prescribed chemotherapies included combinations of fluoropyrimidines, irinotecan and oxaliplatin, with 54% of patients receiving oral monotherapy with capecitabine. Clinical interventions undertaken during the telemedicine consultations are shown in Table 1.

## Discussion

Our results demonstrate that telemedicine can be utilised for the assessment and management of older adults with cancer living in a developing country. Importantly, this was a vulnerable population with low previous exposure to technology and with a limited ownership of mobile devices and access to the Internet. Additionally, we demonstrate that telemedicine can be utilised to undertake complex assessments and interventions in this setting, including a geriatric assessment, an assessment of the toxicity of oncological treatments and the prescription of cancer therapy, including cytotoxic chemotherapy. As far as we know, this is the first demonstration of the feasibility of implementing telemedicine in geriatric oncology in a low and middle-income country (LMIC).

The COVID-19 pandemic created barriers to oncology care by leading to canceled, missed or delayed appointments, incomplete assessments and delay in patient treatment [10]. Therefore, as in many hospitals around the world, we were forced to pivot towards the use of telemedicine. Our centre was converted into a COVID-19 hospital in March 2021, leading to the closure of inpatient services for cancer care and to a halt in the admission of new patients. To adapt to this emergency, we attempted to continue providing multidisciplinary geriatric oncology care using telemedicine, as happened in many geriatric oncology clinics [11]. Institutions in the United States, such as the University of Rochester have also reported their experience with the adoption of telemedicine for geriatric assessment in response to the COVID-19 pandemic [12, 13]. However, most teleoncology clinics in the United States have utilised dedicated platforms, which may not be available in LMICs, and which may be difficult to access for older individuals without a personal computer. In addition, there are still disparities for accessing devices and high-speed Internet in LMICs, which also limit the use of telemedicine. However, the growth of mobile technology in developing regions of the world is exponential, with smartphones accounting for 63% of all phones in use [14].

During the COVID–19 pandemic, many countries temporarily authorised the utilisation of online platforms with end-to-end encryption for the provision of medical visits. Such platforms include freely available apps like Zoom, WhatsApp, Teams, Google Meet, among many others [15]. In Mexico, there is a lack of legislation regarding the use of telemedicine, with the National Healthcare Law authorising telephonic communications between patients and providers without specifying a specific way in which such contact can occur and without providing details regarding video communication [16]. Our results show that the preferred method for telemedicine consultations was the WhatsApp platform, which is widely available in Latin America and other regions of the world. There are only a few reports of WhatsApp use in telemedicine, although the app is widely used by physicians as it represents an easy and fast method to contact patients. Nayak *et al* [17] showed that WhatsApp is an effective tool for providing education on tobacco, dental issues and oral cancer compared with conventional audio-visual aids. Recently, Gebbia *et al* [18] reported the use of WhatsApp as a tool for the multidisciplinary management of patients with prostate cancer during the COVID-19 pandemic. Our group has also recently reported the results of the implantation of telemedicine in palliative care consultations during the pandemic, showing a high acceptability and usability with WhatsApp communications among patients receiving supportive care. Unfortunately, there is still a lack of evidence of scaled-up, sustained initiatives in LMICs, and long-term funding is needed to move beyond pilots and develop programmes which can become standard of care [14].

This study adds to the literature by showing that complex interventions, including geriatric assessments and follow-up can be performed through these apps in a vulnerable population, such as older adults with low exposure to digital health tools. Previous results have shown that, among vulnerable patients, telemedicine obtains similar results and face-to-face care, with some considering it better due to increased access and availability, which represents a very significant and valuable endpoint for older adults [19, 20]. In addition, we were able to perform other interventions, including chemotherapy prescription, via telemedicine. Previous research, dating back to the early 2000s, has demonstrated that teleoncology can make it possible for patients living in resource-limited settings to access specialised oncology care. The initial experience in the University of Kansas Medical Center showed that videocalls could be utilised to provide treatment to patients living in remote rural areas of the state [21] and this was replicated in other high-income countries [22]. However, before the COVID-19 pandemic, few studies had looked at the use of telemedicine to provide oncology care, particularly for vulnerable populations such as older adults or individuals living in developing countries [23]. A recently published systematic review found that most studies focused on providing supportive care interventions, rather than on cancer care itself [24]. This of course has changed due to the pandemic, leading to organisations such as ASCO to publish standards guiding the implantation of telehealth in Oncology [25].

Our study has limitations. Our sample size was small due to the limited number of new patients seen at our centre during the COVID–19 pandemic, and we only included patients with gastrointestinal malignancies. However, we observed very few failures to communicate, and we believe this would be true in a larger sample. In addition, patients with gastrointestinal malignancies arguably have the highest burden of symptoms and need, and thus demonstrating the feasibility of this intervention in that population is highly relevant. Another limitation is that we did not assess whether the interventions led to improved outcomes in this population due to the lack of a control group which continued to receive in-person care. Patients were treated at a cancer centre with a multidisciplinary team available, which may not be the case in many other centres, thus limiting the generalisability of the results. However, we believe that this model could potentially be expanded and lead to collaborations with other institutions that do not have the necessary resources to provide geriatric oncology care. Finally, while many older adults did not have a device of their own, many could communicate with the team with the help of younger relatives, highlighting the importance of the involvement of caregivers when providing remote care for older individuals.

## Conclusions

The use of telemedicine as part of the provision of cancer services is possible in older adults with cancer living in developing countries and with little previous exposure to digital technology. Our study demonstrates that most older patients can obtain access to smartphones and high-speed Internet connections, and that communication via readily available, free and easy to use instant messaging platforms such as WhatsApp represents an excellent tool for geriatric consultations and for the deployment of complex geriatric oncology care. We strongly believe there is a need to implement user-friendly interventions allowing older adults to have easy access to technology without resorting to overly complicated platforms. Healthcare centres and policy makers across the world, but particularly in developing countries, should strive to make access to telemedicine more widespread using readily available platforms, particularly for vulnerable populations such as older adults with cancer.

## Conflicts of interest

The authors have no conflicts of interest to disclose.

## Disclosure of results at meetings

This research was presented in poster format at the 2022 MASCC Annual Meeting. It has not been previously published as a preprint.

## Funding declaration

No funding was received for the current study.

## Figures and Tables

**Table 1.** Clinical interventions undertaken during telemedicine consultations (*n* = 167).

Type of intervention	Visits (*n*)	%
Geriatric assessment	135	80
Review of laboratory results	117	70
Chemotherapy prescription	53	32
Recording of patient’s weight	82	49
Review of imaging studies	42	25
Provision of supportive care recommendations or referrals to supportive/palliative care	34	20
Referral to radiotherapy	3	2
